# SEOM-GEMCAD-TTD clinical guidelines for the adjuvant treatment of colon cancer (2023)

**DOI:** 10.1007/s12094-024-03559-5

**Published:** 2024-06-24

**Authors:** Carles Pericay, Clara Montagut, Juan José Reina, Marcos Melian, Julia Alcaide, Noelia Tarazona, Ana Ruiz-Casado, Encarnación González-Flores, Begoña Graña, Cristina Grávalos

**Affiliations:** 1 Medical Oncology Department, Hospital University, Mútua de Terrassa, Barcelona, Spain; 2 Medical Oncology Department, Hospital del Mar, Barcelona, Spain; 3 Medical Oncology Department, Hospital University, Virgen Macarena, Seville, Spain; 4 Medical Oncology Department, IVO, Valencia, Spain; 5 Medical Oncology Department, Hospital University, Regional y Virgen de la Victoria, Málaga, Spain; 6 Medical Oncology Department, Hospital Clínico University de Valencia, Valencia, Spain; 7 Medical Oncology Department, H.U. Puerta de Hierro, Madrid, Spain; 8 Medical Oncology Department, Hospital University, Virgen de las Nieves, Granada, Spain; 9 Medical Oncology Department, Complexo Hospitalario Universitario, A Coruña, Spain; 10 Medical Oncology Department, Instituto de Investigacion Sanitaria Imas12, Hospital Universitario 12 de Octubre, Madrid, Spain

**Keywords:** Colorectal cancer, Localized disease, Systemic treatment, Guidelines

## Abstract

Colorectal cancer (CRC) has a 5-year overall survival rate of over 60%. The decrease in the rate of metastatic disease is due to screening programs and the population’s awareness of healthy lifestyle. Similarly, advancements in surgical methods and the use of adjuvant chemotherapy have contributed to a decrease in the recurrence of resected disease. Before evaluating a patient’s treatment, it is recommended to be discussed in a multidisciplinary tumor board. In stage II tumors, the pathologic characteristics of poor prognosis must be known (T4, number of lymph nodes analyzed less than 12, lymphovascular or perineural invasion, obstruction or perforation, poor histologic grade, presence of tumor budding) and it is mandatory to determine the MSI/MMR status for avoiding administering fluoropyridimidines in monotherapy to patients with MSI-H/dMMR tumors. In stage III tumors, the standard treatment consists of a combination of fluoropyrimidine (oral or intravenous) with oxaliplatin for 6 months although the administration of CAPOX can be considered for 3 months in low-risk tumors. Neoadjuvant treatment is not consolidated yet although immunotherapy is achieving very good preliminary results in MSI-H patients. The use of ctDNA to define the treatment and monitoring of resected tumors is only recommended within studies. These guidelines are intended to help decision-making to offer the best management of patients with non-metastatic colon cancer.

## Incidence and epidemiology

Colorectal cancer (CRC) is the third most commonly diagnosed cancer globally (1.926.425 cases in 2022) but ranks second in terms of mortality (904.019 deaths in 2022), representing one in ten cancer cases and deaths [1]. In Spain, CRC is the most diagnosed cancer with 43.370 new cases in 2022 (28.706 colon cancer cases and 14.664 rectal cancer cases).

Incidence is higher in males, and this sex disparity increases in left colon [2]. The risk of CRC escalates rapidly with age and 60% of cases are diagnosed between 50 and 74 years. A worrisome rising incidence in younger adults aged less than 50 years has been observed in some countries, happening specially in left-sided and rectal tumors. Regarding anatomic location, the prevalence of right colon cancer (42%) is on the rise compared to left CRC (51%) [3].

Approximately 70% of cases arise sporadically, 25% have a family history of CRC and 5% are attributable to hereditary syndromes due to germline deleterious genetic variants that cause known hereditary diseases, such as Lynch syndrome and familial adenomatous polyposis [4]. Colon cancer is highly preventable, through avoidance of several very well-known risk factors: red and processed meat, smoking, excessive alcohol, physical inactivity and overweight. Interestingly, men have a higher vulnerability to environmental risk factors [2].

Large differences in incidence, mortality, and stage distribution have been described among European countries. There is a tenfold variation in CRC incidence rates by world regions being incidence rates higher in developing countries. Most screen-detected CRC are diagnosed at stages I-II while most non-screen detected are diagnosed at stages III-IV [5]. Currently, 5-year overall and CRC-specific survival range from 57.5 to 83.4% and 65.7 to 89.2%, respectively in Europe [5].

As a screening test for average-risk population, colonoscopy/sigmoidoscopy and fecal occult blood test have demonstrated a lower incidence and CRC-related mortality. However, recently colonoscopy has only shown a decrease in incidence and not in mortality in a large European randomized clinical trial [6].

In the last European Council Recommendation on cancer screening 2022, “quantitative fecal immunochemical testing (FIT) is considered the preferred screening test, instead of fecal occult blood, for referring individuals for follow-up colonoscopy between 50 and 74 years old, and endoscopy may be adopted as a primary tool to implement combined strategies”. They also consider that “quantitative information from FIT results might be used on the basis of further research with a view to implement risk-tailored strategies, introducing thresholds defined per sex, age, and earlier test results” [7].

### Recommendations

- CRC screening is recommended for average-risk population from 50 to 74 years, since it decreases CRC incidence and mortality (III, B).
- Non-colonoscopic test (FIT) is recommended, with an optimal frequency of 1 or 2 years and no later than every three years (I, B). In case of performing colonoscopy (I, B), the repetition interval will be ten years or earlier depending on findings.
- Healthy lifestyle is recommended, avoiding red and processed meat, smoking, alcohol and sedentarism (III, B)

## Methodology

This guideline is based on a systematic review of relevant published studies and with the consensus of ten oncologist experts in treatment from two Spanish digestive cooperative groups (Multidisciplinar Spanish Group of Digestive Tumors, GEMCAD, and Spanish Group for the Treatment of Digestive Tumors, TTD), the Spanish Society of Medical Oncology (SEOM), and an external review panel comprising two experts designated by SEOM. The Infectious Diseases Society of America–US Public Health Service Grading System for Ranking Recommendations in Clinical Guidelines has been used to assign levels of evidence and grades of recommendation [8] (Table 1).

**Table 1:** Levels of evidence and grades of recommendation

Levels of evidence
I. Evidence from at least one large randomized, controlled trial of sound methodological quality (low potential for bias) or meta-analyses of well-conducted randomized trials without heterogeneity
II. Small randomized trials or large randomized trials with a suspicion of bias (lower methodological quality) or meta-analyses of such trials or of trials with proven heterogeneity
III. Prospective cohort studies
IV. Retrospective cohort studies or case–control studies
V. Studies without control group, case reports, experts opinions
Grades of recommendation
A. Strong evidence for efficacy with a substantial clinical benefit; strongly recommended
B. Strong or moderate evidence for efficacy, but with limited clinical benefit; generally recommended
C. Insufficient evidence of efficacy or benefit does not outweigh the risk or the disadvantages (adverse events, costs,); optional
D. Moderate evidence against efficacy or for adverse outcome; generally not recommended
E. Strong evidence against efficacy or for adverse outcome; never recommended

### Diagnosis, multidisciplinary tumor board and surgical procedures

Localized colon cancer may present clinically with abdominal pain, rectal bleeding, iron deficiency anemia (the most common finding), asthenia, changes in bowel habit or obstructive symptoms. Up to 20–25% of cases are asymptomatic at diagnosis, being identified by screening test or for other reasons. In these cases, the prognosis is usually better than in those presenting with symptoms [9].

It is important to collect the personal and family history of cancer and premalignant lesions to assess the convenience of referral for genetic counseling. Performance and nutritional status should be documented in the clinical record.

Routine laboratory tests should include a complete blood count, biochemistry with liver/renal function tests and CEA prior to surgery, which is an independent prognostic factor for overall survival [10].

Colonoscopy is the diagnostic method of choice for histologic confirmation. It also allows localization of the tumor with tattooing if necessary and complete evaluation of the colonic mucosa to visualize/resect possible synchronous cancers and/or polyps. In case of stenotic distal colonic tumors that cannot be passed with the colonoscope, CT colonography, intraoperative colonoscopy or delayed colonoscopy after surgery of the primary tumor could be considered.

CT scan of chest/abdomen/pelvis with intravenous and oral contrast is the standard imaging test for colon cancer staging. MRI may be useful in patients with allergy to iodinated contrast, suspected infiltration of surrounding structures or with indeterminate liver lesions. PET/CT is not recommended for initial staging [11].

It is recommended that all patients with a diagnosis of colon cancer be discussed in a multidisciplinary team (MDT) meeting, that will assess whether it is necessary to complete the diagnostic tests and propose an agreed therapeutic approach. Retrospective studies indicate that adequate MDT processes are associated with improved survival for patients with CRC [12].

Endoscopic resection for some early stage tumors and surgical resection for the rest of localized colon cancer are the only curative treatments. The impact of interval from colon cancer diagnosis to surgery on oncological outcome remains unclear, but it is recommended that the curative intent colectomy should be performed without unneeded delay. Complete R0 resection should be the primary goal. At the surgery, a meticulous exploration should be executed and documented in the operative report. The extent of resection depends on the site of the primary lesion and its lymphovascular drainage. The number of lymph nodes evaluated at the time of resection has been associated with survival and guides postoperative management such as chemotherapy administration, therefore the lymphadenectomy should be as complete as possible. In case of clinical T4b tumors, en bloc resection of adjacent organ-affected must be carried out.

Location in the colon marks the technics: right hemicolectomy for caecum, ascendent or transvers cancers, left hemicolectomy for spleen angle and left-sided tumors, sigmoidectomy for sigmoid tumors and total colectomy for multicenter tumors. Laparoscopy approaches have demonstrated equivalent oncological outcomes with decreased length of hospital stay and postoperative complications [13].

#### Recommendations

- Complete blood count, biochemistry, CEA, colonoscopy and CT of chest, abdomen and pelvis are recommended. However, PET/CT is not recommended in the initial staging of localized colon cancer (II, A).
- Multidisciplinary team discussion is recommendable for all patients (IV, B).
- If feasible, the entire colorectal mucosa should be evaluated for synchronous pathology (III, A)
- When expertise is available, laparoscopy approach for elective colectomy for colon cancer is preferred (II, A)

### Staging and pathologic report

The definitive staging of localized colon cancer should be conducted after surgery. The pathologic stage must be reported following the Union for International Cancer Control (UICC) tumor, node, metastasis (TNM) classification, 8th edition (Table 2) [14].

**Table 2:** UICC TNM staging (8th edition) classification for colon and rectal cancer

T—Primary tumour
TX Primary tumour cannot be assessed
T0 No evidence of primary tumour
Tis Carcinoma in situ: Invasion of lamina propria
T1 Tumor invades submucosa
T2 Tumor invades muscularis propria
T3 Tumor invades subserosa or into non-peritonealised pericolic or perirectal tissues
T4 Tumor directly invades other organs or structures and/or perforates visceral peritoneum
T4a Tumor perforates visceral peritoneum
T4b Tumor directly invades other organs or structures
N—Regional lymph nodes
NX Regional lymph nodes cannot be assessed
N0 No regional lymph node metastasis
N1 Metastasis in 1–3 regional lymph nodes
N1a Metastasis in 1 regional lymph node
N1b Metastasis in 2–3 regional lymph nodes
N1c Tumor deposit(s), i.e., satellites, in the subserosa, or in non-peritonealised pericolic or perirectal soft tissue *without* regional lymph node metastasis
N2 Metastasis in 4 or more regional lymph nodes
N2a Metastasis in 4–6 regional lymph nodes
N2b Metastasis in 7 or more regional lymph nodes
M—Distant metastasis
M0 No distant metastasis
M1 Distant metastasis
M1a Metastasis confined to one organ [liver, lung, ovary, non-regional lymph node(s)] without peritoneal metastases
M1b Metastasis in more than one organ
M1c Metastasis to the peritoneum with or without organ involvement

Stage	Tumor	Nodes	Metastasis
0	Tis	N0	M0
I	T1, T2	N0	M0
IIA	T3	N0	M0
IIB	T4a	N0	M0
IIC	T4b	N0	M0
IIIA	T1-T2	N1/N1c	M0
IIIA	T1	N2a	M0
IIIB	T3-T4a	N1/N1c	M0
IIIB	T2-T3	N2a	M0
IIIB	T1-T2	N2b	M0
IIIC	T4a	N2a	M0
IIIC	T3-T4a	N2b	M0
IIIC	T4b	N1-N2	M0
IVA	Any T	Any N	M1a
IVB	Any T	Any N	M12
IVC	Any T	Any N	M1c

aTis includes cancer cells confined within the mucosal/lamina propria (intramucosal) with no extension through the muscularis mucosae into the submucosa

bInvades through to visceral peritoneum to involve the surface

cDirect invasion in T4b includes invasion of other organs or segments of the colorectum by way of the serosa, as confirmed on microscopic examination, or for tumors in a retroperitoneal or subperitoneal location, direct invasion of other organs or structures by virtue of extension beyond the muscularis propria

dTumour that is adherent to other organs or structures, macroscopically, is classified cT4b. However, if no tumour is present in the adhesion, microscopically, the classification should be pT1–3, depending on the anatomic depth of wall invasion

eTumour deposits (satellites) are discrete macroscopic or microscopic nodules of cancer in the pericolorectal adipose tissue’s lymph drainage area of a primary carcinoma that are discontinuous from the primary and without histologic evidence of residual lymph node or identifiable vascular and/or perineural invasion. If a vessel wall is identifiable on H&E, elastic or other stains, it should be classified as venous invasion (V1/2) or lymphatic invasion (L1). Similarly, if neural structures are identifiable, the lesion should be classified as perineural invasion (PNI). The presence of tumour deposits does not change the primary tumour T category, but changes the node status (N) to pN1c if all regional lymph nodes are negative on pathologic examination

The standard pathologic report should include the following [15]:

- a detailed description of the specimen's morphology;
- information about the surgical procedure performed;
- clear definition of the tumour location and size;
- identification of macroscopic tumor perforation, if present or absent;
- histologic type and grade of the cancer with histologic subtypes and binary classification (low- or high-grade tumor);
- depth of infiltration (T stage);
- number of evaluated lymph nodes and number of positive nodes (N stage). It is recommended to examine at least 12 lymph nodes to accurately stage colon cancers according to the AJCC and College of American Pathologists [16].
- Involvement not due to contiguity of other organs (M stage);
- status of the margins (proximal, distal, radial, and mesenteric);
- presence of lymphovascular invasion; intramural or extramural.
- (EMVI), perineural invasion, tumor deposits, and tumor budding*;
- mismatch repair (MMR) or microsatellite instability (MSI) status of the tumor, assessing using immunohistochemistry and/or molecular biology or both.

*Tumor deposits are proposed as an independent negative prognostic factor and high tumor budding has been identified as a new poor prognostic factor in colon cancer [17].

### Biomarkers

Dihydropyrimidine-dehydrogenase (DPD), encoded by the DPYD gene, is the critical enzyme implicated in fluoropyrimidines (FP) metabolism. The partial or complete deficiency of this enzyme has been associated with greater toxicity from FP. The AEMPS (Spanish Agency for Medicines and Health Products) recommends genotyping patients before treatment with FP [18]. In addition, a consensus of experts of the Spanish Pharmacogenetics and Pharmacogenomics Society (SEFF) and SEOM, establishes recommendations for the implementation of genotype DPD deficiency in patients candidates to FP [19].

Universal MMR or MSI testing is recommended for all newly diagnosed patients with localized colon cancer, on resected specimen or initial diagnostic biopsy. Deficient mismatch repair deficiency (dMMR) status can be identified through immunohistochemistry detecting loss of MMR protein expression (MLH1, MSH2, MSH6, or PMS2) or through polymerase chain reaction (PCR) assays of MSI status (high microsatellite instability (MSI-H)). Nowadays, this determination serves two purposes: prognosis and prediction of adjuvant benefits and potential genetic predisposition [20]. However, it could be also relevant for selecting patients with dMMR/MSH-I tumor for neoadjuvant immunotherapy if it becomes a standard of care in a near future.

Genetic markers such as *RAS* and BRAF mutations are not advisable for routine evaluation of recurrence risk in non-metastatic patients, although recent data suggest a prognostic role of KRAS exon 2- and BRAFV600E mutations [21]. There are other biomarkers such as gene signatures, Immunoscore, and postoperative circulating tumor DNA (ctDNA) that have demonstrated some benefit in determining the risk of recurrence and could be considered in specific cases. However, they are not routinely implemented.

Immunoscore is a strong predictor of time to recurrence, overall survival (OS), and disease-free survival (DFS), independently of patient age, sex, MMR/MSI status, and other prognostic factors. Immunoscore has been recently validated in a large prospective cohort of over 2500 patients with TNM stage I-III. However, its role in predicting chemotherapy benefit is uncertain, and firm evidence of its prognostic role in stage II-only datasets is currently lacking [22].

In addition, ctDNA is a promising tool under investigation to identify patients at high risk of recurrence after primary tumor resection, and it will be addressed in a specific section.

#### Recommendations

- The risk of relapse after colon cancer resection should be assessed by integrating TNM staging, MMR/MSI status, and the number of lymph nodes sampled (III A).
- Other clinicopathological features, such as histologic subtype and grading, lymphatic or venous or perineural invasion, lymphoid inflammatory response, involvement of resection margins, and serum CEA levels, should be considered to define the risk assessment for stage II tumors (III A).
- MSI/MMR status is the only validated molecular marker used in adjuvant decision-making and should be determined in all stages (IV A).
- Gene-expression signatures and Immunoscore are not recommended for routine practice due to a lack of predictive value for chemotherapy benefit but may be considered in conjunction with TNM scoring to adjust the chemotherapy decision-making process in stage II and even in low-risk stage III patients (III C).
- Implementation of genotype and/or phenotype testing for DPD deficiency is mandatory in patients who are candidates to receive FP (III, A).

### Adjuvant treatment in stage II

**Fig. 1:**
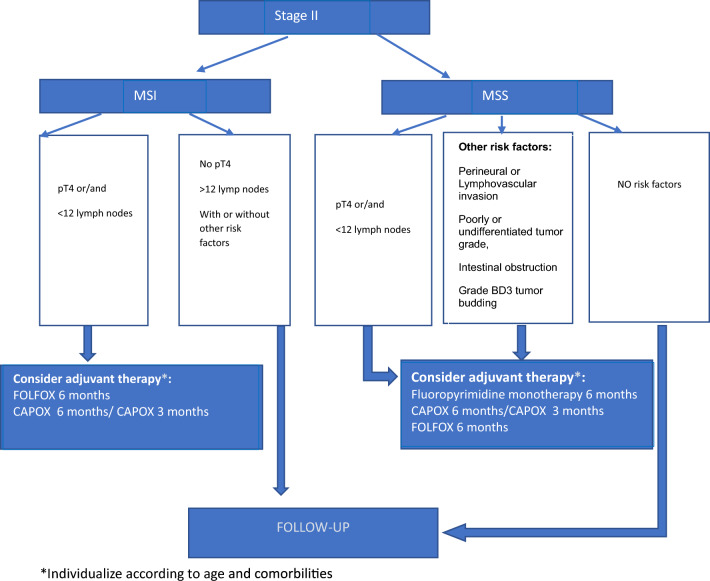
Stage II. Adjuvant treatment recommendations. *Individualize according to age and comorbilities

Adjuvant chemotherapy (ACT) is not routinely recommended for all patients with stage II colon cancer (Fig. 1). A systematic review and meta-analysis found that ACT resulted in a small DFS advantage, but no increase in OS, compared with surgery alone. In a recent meta-analysis, the estimated 5-year DFS for patients with stage II colon cancer treated with ACT was 79.3%, compared with 81.4% for patients who did not receive ACT [23].

ACT should not routinely be offered to patients who are at low risk for recurrence. A small number of retrospective cohort studies with high variability depending on the underlying patient population, characterize the limited data for patients with low-risk stage II colon cancer.

The ESMO Clinical Practice Guidelines 2020 establishes major and minor clinicopathological factors that impact on the risk of relapse on stage II colon cancer. The presence of major factors including pT4 stage, < 12 lymph nodes assessed and perforation confers increased risk of recurrence, while the presence of other additional risk factors as perineural (PNI) or lymphovascular invasión (LVI), poorly or undifferentiated tumor grade, intestinal obstruction or preoperative CEA > 5 ng/ml, is less significantly associated with risk of relapse [24].

The ASCO Guideline 2022 defines higher risk of recurrence as stage IIB and stage IIC colon cancer (i e., pT4, lesions either penetrating visceral peritoneum or invasive of surrounding organ, respectively) and high-risk stage IIA (pT3) with sampling of fewer than 12 lymph nodes in the surgical specimen, PNI or LVI, poorly or undifferentiated tumor grade, intestinal obstruction, tumor perforation, and/or grade BD3 tumor budding [25]. The International Tumor Budding Consensus Conference concluded that specimens with grade BD3 budding are associated with an increased risk of recurrence in stage II CRC [26].

For inadequate surgical margins, low- to very low-quality evidence was found for the effect of ACT versus surgery alone.

All studies included in the systematic review of the medical literature find a positive effect of ACT on OS in patients with pT4 tumors and/or fewer than 12 sampled lymph nodes. Based on this data, ACT should be offered to these patients [27, 28]. For pT3 tumors, the number of risk factors should be considered as part of the shared decision-making process, because the presence of risk factors (including BD3 budding) may increase the risk of recurrence. ACT may be offered to patients with stage IIA (pT3) colon cancer with high-risk features.

There is not enough evidence to routinely recommend the addition of oxaliplatin to fluoropyrimidine-based chemotherapy for patients with high-risk stage II colon cancer based in exploratory analyses of the MOSAIC trial [29].

The presence of MSI-H/dMMR in localized disease confers better prognosis and less benefit to adjuvant fluoropyrimidine-only chemotherapy. FP monotherapy is not routinely recommended [30]. For pT4 or pT3 plus other high-risk features (with the exception of poor differentiation) tumors, oxaliplatin-containing chemotherapy may be individually considered. It is based on a subgroup analysis of four randomized trials from the IDEA collaboration.

The adjuvant oxaliplatin-containing chemotherapy may be offered for a duration of 3 or 6 months, after a discussion with the patient of the potential benefits and risks [31].

#### Recommendations

- Adjuvant chemotherapy (ACT) should not routinely be offered to all patients with stage II colon cancer (I, A).
- ACT should not routinely be offered to patients who are at low risk for recurrence (III, C).
- ACT should be offered to patients with stage II, pT4 (including perforation) colon cancer and/or fewer than 12 lymph nodes in the surgical specimen, with a discussion of the potential benefits and risks of harm associated with ACT (III, C).
- ACT may be offered to patients with stage T3 colon cancer with high-risk features, including perineural or lymphovascular invasion, poorly or undifferentiated tumor grade, intestinal obstruction, and/or grade BD3 tumor budding (III, B).
- There is insufficient evidence to routinely recommend the addition of oxaliplatin to fluoropyrimidine-based chemotherapy for patients with high-risk stage II colon cancer (III, C).
- Adjuvant fluoropyrimidine-only chemotherapy in stage II is not routinely recommended for patients with dMMR or MSI-H tumors (II, A).
- In patients who are candidates for adjuvant doublet chemotherapy, oxaliplatin-containing chemotherapy may be offered for a duration of 3 (CAPOX) or 6 months (CAPOX or FOLFOX), after a discussion with the patient of the potential benefits and risks of harm associated with the options for treatment duration (II, B).

### Adjuvant treatment in stage III

**Fig. 2:**
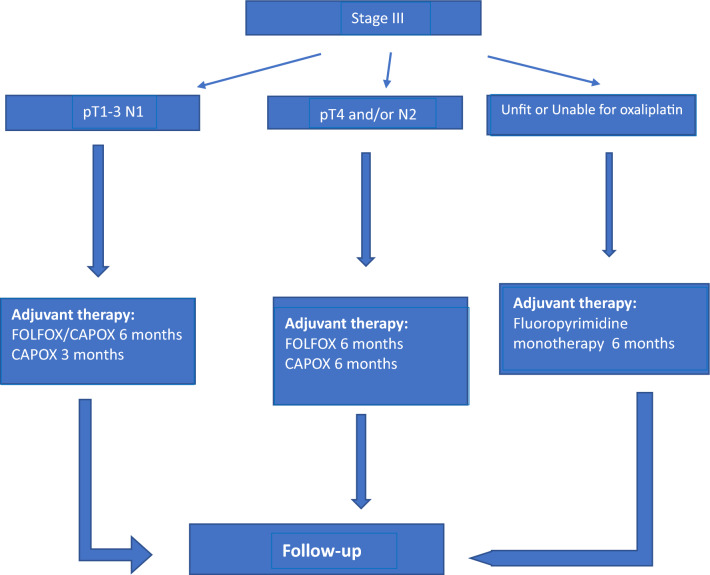
Stage III. Adjuvant treatment recommendations

Standard ACT for patients with stage III colon cancer consists of the combination of fluoropyrimidine (5FU or capecitabine) and oxaliplatin (Fig. 2). The benefit of adding oxaliplatin versus fluoropyrimidine monotherapy has been established on the basis of three major trials: MOSAIC [30–32], NSABP C-07 [33] and NO16968 (XELOXA) [34, 35].

Both the MOSAIC and XELOXA studies found a significant increase in DFS and OS when oxaliplatin was associated with a 6-month schedule based on continuous infusion 5FU (FOLFOX4) or capecitabine (CAPOX), respectively. Further follow-up confirmed the impact on OS in both studies, with a relative decrease in the risk of death of 20% and 17%. In MOSAIC study, the benefit was more prominent for patients with N2 tumors [32].

In the NSABP C-07 trial, both the control (FULV) and experimental (FLOX) arms included bolus 5FU and leucovorin. After eight years of follow-up, there was a significant increase in DFS in stage III patients treated with FLOX and a trend toward improved OS, which did not reach statistical significance. The lack of statistically significant impact on OS, and the greater toxicity of the FLOX regimen, have led to its use as a standard adjuvant regimen not being recommended [33].

Regarding the optimal duration of treatment, the IDEA study [36] compared the administration of FOLFOX or CAPOX for 3 or 6 months. Although a shorter duration led to less toxicity, the study did not meet the pre-specified 3-month non-inferiority target, which was based on accepting at most a loss of half of the benefit obtained by adding oxaliplatin in the MOSAIC study. Nevertheless, DFS rates were similar between both arms (3-year DFS: 74.6% for 3 months vs 75.5% for 6 months) and the absolute difference in 5-year OS was only 0.4%.

In a pre-planned analysis, an interaction effect of the regimen used was observed, such that in patients treated with CAPOX, although statistically significant non-inferiority of 3 months for DFS was not demonstrated, DFS rates were numerically higher for 3 months. In contrast, in those treated with FOLFOX, superior DFS was seen for 6 months versus 3 months. It should be mentioned that the allocation of the scheme was not randomized, but was at the choice of the investigator.

In addition, an exploratory analysis was performed, based on risk group: low risk (T1-T3 N1) and high risk (T4 and/or N2). In the low-risk subgroup, significant non-inferiority at 3 months versus 6 months could not be confirmed, although in those treated with CAPOX, DFS rates were even numerically higher for 3 months. In high-risk patients, DFS was superior for those who received 6 months of adjuvant therapy, especially when the scheme of choice was FOLFOX. It should be noted that the analysis by these risk subgroups, grouping the T and N categories, was a post-hoc analysis, and that the interaction between duration and risk group was not significant.

Recently, a combined analysis of the ACCENT and IDEA databases [37] observed a reduction in DFS and OS with early discontinuation of adjuvant treatment, i.e., before receiving 75% of cycles, as opposed to early discontinuation of oxaliplatin alone (before 10 cycles of FOLFOX or 7 cycles of CAPOX), which did not impact survival. The exception was the low-risk subgroup treated with CAPOX, where DFS was similar regardless of early discontinuation or not. Patients who received less than 50% of planned cycles of oxaliplatin had significantly shorter DFS and OS.

There are conflicting data on the timing of adjuvant chemotherapy, although it is generally accepted that it should be initiated as soon as possible after the patient has recovered from surgery, and ideally no later than 8 weeks after surgery [38].

It is not advisable to reduce the dose or limit the body surface area to 2 m^2^ in obese patients [39].

Neither irinotecan [40, 41], cetuximab [42, 43], bevacizumab [44, 45] nor celecoxib [46] have led to an improvement in the adjuvant treatment of colon cancer, so their use is not recommended.

#### Recommendations

- ACT of patients with stage III colon cancer should consist of a combination of fluoropyrimidine and oxaliplatin (*I. A).*
- The duration of ACT can be adapted according to the risk group and the scheme used: 3 months of CAPOX for low-risk patients (T3 and N1) and 6 months of CAPOX for high-risk cases (T4 and/or N2). In case FOLFOX is used, a duration of 6 months is recommended, especially in high-risk cases. In high-risk cases requiring discontinuation of oxaliplatin due to toxicity, it is advisable to maintain fluoropyrimidine until completing 6 months, in particular in those treated with FOLFOX (*II. B).*
- The initiation of ACT before 8 weeks after surgery is strongly recommended (*II. A).*
- It is indicated to administer the full dose of ACT in obese patients (*II. A).*
- For patients unable to tolerate oxaliplatin, schemes with FP in monotherapy (based on 5FU in continuous intravenous infusion or oral capecitabine) can be used. In this case, the duration of ACT should be 6 months (*I. A).*

### Adjuvant chemotherapy in older patients

Patients over 65 years old account for more than 50% of CRC diagnosis and are reported to have a worse survival [47]. Elderly patients are under-represented in prospective adjuvant studies and tend to receive chemotherapy less frequently because of higher impaired organ function and multiple comorbidities [48].

However, retrospective and population studies have found that ACT is beneficial in stage III CRC older patients. An analysis from the Surveillance, Epidemiology and End Results database (SEER) database including 69,946 stage II/III patients ≥ 70 years diagnosed with CRC between 2004 and 2012 showed that stage III patients receiving treatment have a 35.8% lower cancer-specific mortality rate (HR = 0.642, 95% CI 0.620–0.665, *P* < 0.001) compared with those who did not receive chemotherapy after adjusting for confounding variables.

Surprisingly, stage II CRC patients aged 70 or older receiving ACT exhibited a 44.6% higher 5-year cancer-specific mortality rate comparing with no treatment (5-year cancer-specific survival 82.0% and 72.4%, respectively *P* < 0.001) [49]. However, these results should be interpreted with caution as only 3.053 (8.7%) out of 35.099 stage II patients were treated with ACT and there was no information about high-risk factors. A metanalysis including more than 20,000 patients ≥ 70 years showed there was no significant difference in OS rate between stage II patients who received ACT vs no ACT (*P* = 0.09), while stage III patients who received adjuvant chemotherapy had a significantly better OS (*P* < 0.001) in all age subgroups (≥ 70, ≥ 75, and ≥ 80 years) than patients not treated [50].

Actually, the benefit of adding oxaliplatin to 5-FU/LV regimens is not well established in patients ≥ 70 years [51]. Recent retrospective data analysis of more than 41.000 elderly patients with stage III showed a survival benefit of multi-agent chemotherapy (MAC) when compared with single-agent chemotherapy (SAC) in the 70–75 age subgroup while MAC seemed to have similar efficacy as SAC in those aged > 76 years [52]. Also, a subgroup analysis of the phase III TOSCA (part of the IDEA trial) found that there was no difference in time to tumor recurrence between patients ≥ 70 years vs < 70 years when treated with oxaliplatin (*P* = 0.082), although a greater proportion of dose reductions (46.7% vs 41.4%, *P* = 0.018) and treatment interruptions (26.1% vs 19.3%, *P* < 0.001) were performed in older patients [53].

Ultimately, management decisions in the care of older adults with CRC, including those with dMMR/MSI-H stage II and III tumors, must be made through shared decision-making with the patient with consideration for the patient’s functional status, comorbidities, goals of care, social support, as well as toxicities and possible effect on quality of life (QoL) [54].

#### Recommendations

- The lack of evidence of a clear benefit of treatment in elderly patients with stage II CRC should lead us to individualize treatment according to age and comorbidities (III, B)
- In patients ≥ 70 years old affected with stage III CRC the benefit of ACT with 5-FU/LV/capecitabine based regimens is well stablished (I, A)
- The addition of oxaliplatin to 5-FU/LV in the elderly population is controversial and should be carefully planned based on shared decision-making with each patient (II, B)

### Neoadjuvant treatment

Neoadjuvant treatment is being tested as an emerging alternative for the treatment of locally advanced colon cancer (LACC) patients. Neoadjuvant treatment could potentially offer some benefits including tumor shrinkage, eradication of micrometastases, and prevention of tumor cell shedding during surgery. The potential disadvantages are increased post-operative morbidity and overtreatment. In this regard, locoregional staging of colon cancer with current radiologic methods is not accurate, especially for cT3 vs cT4 and for nodal status. The training of radiologists and optimization of the radiologic methods are crucial to correctly select patients for neoadjuvant treatment [55, 56].

The Fluorouracil, Oxaliplatin and Targeted Receptor pre-Operative Therapy (FOxTROT) is the first randomized clinical trial that evaluated the efficacy of neoadjuvant chemotherapy (NAC) in LACC [57]. Patients with radiologically staged T3-4, N0-2, M0 colon cancer were randomly allocated (2:1) to 6 weeks oxaliplatin-fluoropyrimidine preoperatively plus 18 weeks postoperatively (NAC group) or 24 weeks post-operatively (control group). Patients with RAS-wildtype tumors could also be randomly assigned 1:1 to receive panitumumab or not during NAC. The FOxTROT included 699 patients and reached its primary endpoint: fewer NAC than control patients had residual or recurrent disease within 2 years (16.9% vs 21.5%; *P* = 0.037). NAC produced marked T and N downstaging and histologic tumor regression (all *P* < 0.001), which correlated strongly with recurrence-free survival. Panitumumab did not enhance the benefit from NAC. Importantly, little benefit from NAC was seen in dMMR tumors. Of note, 25% of patients had an incorrect radiologic staging (low-risk stage II tumors) in the control group, confirming the difficulty of accurate radiologic assessment in LACC patients and the risk of overtreating patients with NAC strategy.

#### Recommendations

- Neoadjuvant treatment in LACC is not recommended as a standard and need to be discussed with the patient (I, C)

### Mismatch repair (MMR) and microsatellite instability (MSI) status. Immunotherapy in localized MSI tumors

Mismatch repair /microsatellite instability status in localized colon cancer patients has two objectives: to determine potential genetic predisposition and to characterize the prognosis and prediction of benefit of adjuvant treatment. MSI/MMR status defines a subgroup of patients with a better prognosis and less expected benefit from chemotherapy. In particular, MSI/MMR status identifies a small (10%-15%) subset of stage II patients who are at a very low risk of recurrence and in whom the benefits of FP in monotherapy have not been demonstrated and thus they should not be indicated [58–60].

Given the positive results of immunotherapy in MSI-H/dMMR tumors in the metastatic setting, immune checkpoint inhibitors (ICI) are being evaluated in ongoing randomized clinical trials in patients with MSI-H early colon cancer as neo/adjuvant treatment. In the neoadjuvant setting, immunotherapy is showing very promising results. The NICHE trial was a phase II exploratory study that included 60 patients with radiologic stage I-III colon cancer (30 dMMR and 30 pMMR) treated with ipilimumab and nivolumab. Treatment was well tolerated and all patients underwent radical resections without delays. Major pathologic response was achieved in 97% and 29% of dMMR and pMMR tumors, respectively [61]. NICHE-2 trial was a single arm phase II trial that included 112 dMMR tumors ≥ cT3 and/or cN + based on radiologic staging. Treatment was well tolerated and all patients underwent surgery, achieving a major pathologic response in 95% of patients and 67% pCR [62]. Results of the primary endpoint on 3-year DFS are eagerly awaited.

#### Recommendations

- ACT with FP as single agent should not be indicated in MSI-H/dMMR in stage II patients (I, A)
- Consider ACT with oxaliplatin + fluoropyrimidine for high-risk stage II and III MSI-H/dMMR tumors (III, C)
- The complete response rate in patients with MSI-H tumors following ICI in neoadjuvant trials has potential organ-sparing implications, but longer follow-up, more mature data and results from randomized trials are needed (III, C)

### State of ctDNA determination. Implications in treatment decision-making and follow-up

The monitoring of circulating tumor DNA, also referred to as liquid biopsy, is being investigated as a promising tool to identify patients who are at a high risk of recurrence after the surgical removal of the primary tumor [63]. In recent years, numerous retrospective studies have demonstrated that the detection of ctDNA immediately after surgery and at any time during follow-up, known as mutation tracking, is associated with poorer DFS and identifies patients at high risk of relapse in localized CRC [64–66].

The detection of ctDNA precedes radiologic relapse by a median of 8.2 months [67]. However, a significant proportion of patients with detectable ctDNA after surgery do not clear the ctDNA after completing ACT (ranging from 13 to 77% depending on the published series). The detection of ctDNA after completion of ACT is also associated with worse DFS and indicates resistance to standard therapy. These findings suggest that there is a potential need to personalize the treatment for patients with minimal residual disease (MRD) to more efficiently eradicate the disease, reducing the risk of relapse.

The DYNAMIC study was the first prospective, randomized study to demonstrate that ctDNA could guide therapeutic decisions in patients with stage II CRC. Patients were assigned to ctDNA-guided management versus the standard of care. After a median follow-up of 37 months, a lower percentage of patients in the ctDNA-guided management group received ACT compared to the standard management group (15% vs. 28%; RR, 1.82). ctDNA-guided management was noninferior to standard management at 2-year DFS (93.5% vs. 92.4%) [68].

Remarkably, a study conducted across longitudinal sampling during post-treatment surveillance showed that serial assessment of ctDNA, instead of a single time point, enhances the sensitivity of the overall analysis while maintaining a high level of specificity [67]. This heightened accuracy of serial ctDNA assessment introduces exciting possibilities for ctDNA analysis beyond evaluating MRD after surgery. One potential application is the longitudinal allocation of imaging resources for recurrence surveillance based on risk stratification. Data suggests that radiologic surveillance could be reduced in low-risk patients (ctDNA-negative) with minimal impact on outcomes. As this subgroup constitutes the majority of patients, this approach would effectively lower surveillance costs. Conversely, for high-risk patients (ctDNA-positive), there is an opportunity to intensify imaging immediately upon ctDNA detection.

Although the published results so far are promising, the SEOM-GEMCAD-TTD panel believes that there is currently insufficient evidence to recommend routine use of ctDNA assays outside of a clinical trial. The outcome of these trials will likely define the definitive role of ctDNA in the decision-making process for adjuvant treatment and surveillance.

#### Recommendation

- The use of ctDNA in the management of localized colon cancer is not recommended outside of clinical trials (II, C)

## Follow-up, long-term implications and survivorship

The main objective of follow-up is to improve survival through an early detection of relapse. Usefulness of CEA is widely accepted [69] and CT scans seem to be also necessary for early detection. The frequency of visits will depend on the time elapsed from surgery and could also depend on the risk of relapse.

80% of recurrences will be detected in the first 3 years after the diagnosis, which could support a more intensive follow-up during 3 years. Though is commonly accepted that follow-up is stopped at 5th year, late relapses can occur up to 8 years. After 8 years the notion of cure is appropriate.[70] Colonoscopies should be included in the follow-up since metachronous cancer can be detected with an incidence of 0.7%.

### Survivorship

Two crucial points in CRC survivorship are the prevention or early diagnosis of new metachronous cancer and the promotion of a healthy lifestyle. In addition, it is important to address the care of cancer and treatment consequences, such as neuropathy, cardiotoxicity, and cancer-related fatigue.

Healthy lifestyle includes smoking cessation, engaging in regular exercise and keeping body mass index into the recommended limits. Large cohort studies have shown an association between physical activity and lower rates of both cancer-related mortality and overall mortality [71]. Dietary patterns including fruits, vegetables, poultry and fish with less red meat and concentrated sweets have shown improved outcomes [72, 73].

#### Recommendations

- Surveillance after colon cancer treatment leads to earlier detection of recurrences and more surgeries with curative intent. (II, B)
- Colonoscopy at 1 year after surgery. If normal (or no new findings), repeat colonoscopy at 3 years and then every 5 years. (III, B)
- History, physical examination, and CEA every 3 to 6 months for 3 years. Then every 6 months until 5th year. (II, B)
- CT scan of chest/abdomen/pelvis every 6–12 months for 5 years (II, B)
- A healthy lifestyle increasing physical activity should be encouraged for cancer survivors (III, A)
- Following a healthy diet and keeping a healthy body composition should be recommended by oncologists (III, B)

## Ethical approval

Compliance with ethical standards. The current study has been performed in accordance with the ethical standards laid down in the 1964 Declaration of Helsinki and its later amendments.

## Data Availability

Not applicable.
